# 231. Retrospective Comparison of Intravenous Therapy, Oral Therapy, and Lipoglycopeptides for the Treatment of Osteomyelitis

**DOI:** 10.1093/ofid/ofab466.433

**Published:** 2021-12-04

**Authors:** Alex Stumphauzer, Ryan P Moenster, Travis W Linneman

**Affiliations:** 1 VA St. Louis Health Care System, St. Louis, Missouri; 2 University of Health Sciences and Pharmacy/VA St. Louis HCS, St. Louis, Missouri; 3 St. Louis College of Pharmacy/VA St. Louis Health Care System, St. Louis, MO

## Abstract

**Background:**

The use of oral (PO) antibiotics and lipoglycopeptides are challenging the previous standard of osteomyelitis (OM) treatment, but there is currently a paucity of comparative data between these approaches.

**Methods:**

This retrospective study included patients diagnosed with OM treated with intravenous (IV) antibiotics, PO antibiotics, or lipoglycopeptides between January 1, 2010 and June 1, 2020. Patients in the PO group could receive no more than 14 days of IV antibiotics prior to the PO course, and inclusion into the lipoglycopeptide group required at least 2 doses of drug to be administered. The primary outcome was occurrence of clinical failure within six months of completion of therapy, which was defined as new antibiotics or unplanned surgical intervention for an infection at the same site. Secondary outcomes included in-hospital length of stay (LOS), amputation within 6 months of therapy completion, and incidence of drug and line-related adverse effects. Previous osteomyelitis at index site, surgical intervention as a part of initial management, presence of *Staphylococcus aureus* on culture, utilization of outpatient parenteral antibiotic therapy (OPAT) services (IV group only), and concomitant PO therapy (lipoglycopeptide group only) were included in a bivariate analysis and variables with a *p*-value < 0.2 were included in a multivariate regression model.

**Results:**

The IV group included 257 patients, while the PO and lipoglycopeptide groups included 20 and 15 patients respectively. In the IV group, 89 (35%) of the patients experienced clinical treatment failure compared to 5 (25%) in the PO group and 5 (33%) in the lipoglycopeptide group (p=0.71). Median LOS was significantly shorter in the PO group compared to the IV and LGP groups [1 day (IQR 0-2.5) vs. 7 days (IQR 4-10) and 4 days (IQR 4-9), p=0.003]. No difference between groups was observed for amputation within 6 months or incidence of adverse effects. The only variable included in the multivariate regression model was previous osteomyelitis at index site [OR 1.75, 95% CI (1.07 – 2.87)].

**Conclusion:**

PO and lipoglycopeptide therapy resulted in similar outcomes compared to IV antibiotics. Only previous OM at the same site was identified as an independent risk factor for failure.

**Disclosures:**

**Ryan P. Moenster, Pharm.D., FIDSA**, **AbbVie** (Speaker’s Bureau)**Melinta** (Consultant, Speaker’s Bureau)

